# The role of fish life histories in allometrically scaled food‐web dynamics

**DOI:** 10.1002/ece3.4996

**Published:** 2019-02-21

**Authors:** Stephanie Bland, Fernanda S. Valdovinos, Jeffrey A. Hutchings, Anna Kuparinen

**Affiliations:** ^1^ Department of Biology Dalhousie University Halifax NS Canada; ^2^ Department of Ecology and Evolutionary Biology University of Michigan Michigan; ^3^ Center for the Study of Complex Systems University of Michigan Michigan; ^4^ Institute of Marine Research Flødevigen Marine Research Station His Norway; ^5^ Department of Biological and Environmental Science University of Jyväskylä Jyväskylä Finland

**Keywords:** aquatic ecosystems, bioenergetics model, body size, life histories, niche model

## Abstract

1. Body size determines key ecological and evolutionary processes of organisms. Therefore, organisms undergo extensive shifts in resources, competitors, and predators as they grow in body size. While empirical and theoretical evidence show that these size‐dependent ontogenetic shifts vastly influence the structure and dynamics of populations, theory on how those ontogenetic shifts affect the structure and dynamics of ecological networks is still virtually absent.
2. Here, we expand the Allometric Trophic Network (ATN) theory in the context of aquatic food webs to incorporate size‐structure in the population dynamics of fish species. We do this by modifying a food web generating algorithm, the niche model, to produce food webs where different fish life‐history stages are described as separate nodes which are connected through growth and reproduction. Then, we apply a bioenergetic model that uses the food webs and the body sizes generated by our niche model to evaluate the effect of incorporating life‐history structure into food web dynamics.
3. We show that the larger the body size of a fish species respective to the body size of its preys, the higher the biomass attained by the fish species and the greater the ecosystem stability. We also find that the larger the asymptotic body size attained by fish species the larger the total ecosystem biomass, a result that holds true for both the largest fish in the ecosystem and each fish species in the ecosystem.
4. This work provides an expanded ATN theory that generates food webs with life‐history structure for chosen species. Our work offers a systematic approach for disentangling the effects of increasing life‐history complexity in food‐web models.

## 1 INTRODUCTION

Body size determines key ecological and evolutionary processes during the ontogeny of organisms (Werner & Gilliam, 1984). Ecological interactions, diet breadth, foraging efficiency, reproduction, and mortality, among other processes animating an organism's life, strongly depend on the organism's size (De Roos, Persson, & McCauley, 2003; Werner & Gilliam, 1984; Yodzis & Innes, 1992). Given such dependency, organisms will undergo extensive shifts in resources, competitors, and predators as they grow (Ramos‐Jiliberto, Valdovinos, Arias, Alcaraz, & Garcia‐Berthou, 2011; Werner & Gilliam, 1984). These size‐dependent ontogenetic shifts vastly influence the structure and dynamics of aquatic populations and communities (De Roos et al., 2003; Werner & Gilliam, 1984). For example, “juvenile bottlenecks” influences the structure and dynamics of fish communities, where prey populations compete with the juveniles of their predatory populations exhibiting similar body sizes (Byström, Persson, & Wahlstrom, 1998). Moreover, theoretical work has shown that competitive and predatory (cannibalistic) interactions between different age cohorts drive fish population dynamics (Persson, 1988; van den Bosch, Roos, & Gabriel, 1988; De Roos et al., 2003). However, despite all the empirical and theoretical evidence of the vast impacts of size‐dependent ontogenetic shifts and stage‐structured populations on the population dynamics of interacting species, little theory has been developed on the effects of the size‐dependent ontogenetic shifts and population structure on the structure and dynamics of ecological networks (but see Mougi, 2017). Here, we contribute to develop such theory by expanding the Allometric Trophic Network (ATN; Yodzis & Innes, 1992; Williams & Martinez, 2004b; Williams, Brose, & Martinez, 2007) model to incorporate life‐history structure for fishes (to capture changes in body size across different ages) and evaluate its effect on the structure and dynamics of aquatic food webs.

The study of ecological networks has recently achieved major breakthroughs by recognizing that the ecological functionality of species can be largely attributed to their body sizes (Brose, Jonsson et al., 2006; Otto, Rall, & Brose, 2007). Specifically, a large predator–prey body size ratio appears to be key to stabilizing the dynamics of complex food webs (Brose, Williams, & Martinez, 2006). Through scaling by body size, ATN models have proven successful in explaining the stability, structure, and functioning of ecosystems (Brose, Williams et al., 2006; Dunne, 2006; Williams & Martinez, 2000). Apart from model‐based investigations on the role of body size in food web dynamics, the theory has been further supported by Boit, Martinez, Williams, and Gaedke (2012) who created a remarkably accurate and empirically validated ATN model by incorporating body size that explained 30%–40% of the variation in the seasonal dynamics of the Lake Constance plankton community.

Within the context of food‐web dynamics models in general, and ATN models in particular, species of similar body size have been traditionally lumped together in a single functional group, such that scaling by body size is done with respect to individual body size across the species’ lifespan. This approach stemmed from a need to develop simple models to address generic questions, such as those related to species coexistence (Blondel, 2003). However, for some species, an individual's body size can change by orders of magnitude throughout its life (e.g., fishes; Wootton, 1999). As there are strong correlations between body size and key functional traits, such as metabolic rate (West, 1999), a species’ ecological functionality is likely to change substantially from juvenile to adult life‐history stages. Thus, incorporation of the life‐history structure of species that experience substantial changes in their body size across their lifespan is likely to increase the structural realism of food webs and yield more biologically realistic predictions about their dynamics.

Fishes constitute ideal study species because of their indeterminate growth, which causes them to shift through several ecological niches as they grow (Wootton, 1999). Their body size, diet, exposure to predation, and general ecological functionality changes tremendously from larvae through adult stages, resulting in many species transitioning from the bottom of the food chain to the position of apex predator. For example, during their lives, Atlantic cod (*Gadus morhua*) have the potential to change from being planktivores (as <10 mm, 1–2 g larvae) to apex carnivores longer than 1 m in length and tens of kg in mass within 5–7 years (Brander, 1994; Hutchings & Rangeley, 2011). Another aspect that makes fishes and aquatic food webs particularly interesting systems for studying the role of life‐history structures in food web dynamics is the fact that contemporary life‐history trends toward smaller body sizes and earlier maturity have been documented in many fish species across the world (Audzijonyte, Kuparinen, Gorton, & Fulton, 2013; Hutchings & Baum, 2005). Understanding the impacts that such life‐history changes can have on interacting species, entire ecosystems and sustainable fisheries management warrants for knowledge about the role of fish life histories in food web dynamics.

The present study has two primary objectives. The first is to expand the ATN modeling approach by incorporating simple life‐history structure for the fishes in a generic aquatic ecosystem. The second objective is to evaluate the effect of life‐history structure on food web dynamics. This second objective includes disentangling the effect of increasing food‐web complexity by adding nodes representing the previously ignored life‐history stages from the effect of life‐history dynamics, that is, aging from one life‐history stage to another and reproduction (linkages between life‐history stages). To this end, we use the generic allometrically scaled niche model (Williams & Martinez, 2000) adapted to aquatic food webs (Martinez et al., 2012) to randomly generate scenarios for food webs, within which we introduce life‐history structure to fishes and split the species‐level diets among the life‐history stages. Through systematic simulations, we disentangle the relative impacts of life‐history dynamics from adding life‐history stages by analyzing three types of models: (a) “original” ATN model not including life‐history stages within species, (b) ATN model with “unlinked” life‐history stages that incorporates new nodes but does not connect them via growth and reproduction, and (c) ATN model incorporating life‐history stages that are linked together as a species through aging (hereafter referred to as “growth”) and reproduction. These analyses will provide broadly generalizable insights into the ways in which fish life histories affect their food webs.

## 2 MATERIALS AND METHODS

The theory we develop here consists of generating the topology of life‐history structured food webs which determines the trophic interactions among nodes (i.e., trophic species and fish life‐history stages) and coupling the population dynamics determined by those trophic interactions with life‐history dynamics (fish growth and reproduction). We first describe how we generate the topology of the food webs and then how we link the population dynamics of the species and fish life‐history stages with the life‐history dynamics.

### 2.1 Generation of life‐history structured food webs

We expand the niche model (Williams & Martinez, 2000) to generate networks that incorporate life‐history structures. The niche model uses as inputs the number of species and connectance (i.e., fraction of potential feeding interactions that are realized) and randomly assigns a “niche value” (*n_i_*) to each species from a uniform distribution. This value gives species a hierarchical ranking where they fall relative to each other, which we interpret as relative body size. Species with a low niche value are generally autotrophs, while species with high niche values are more likely to be carnivores. Prey items are assigned to each species from a range centered at a lower niche value, where a larger range indicates a more varied diet. Range size (*r_i_*) is chosen by first drawing a random variable, *x_i_*, from a beta distribution that has been weighted to reflect the desired connectance (*C*) of the web (see Supporting information Appendix for the derivation of β):

(1) $$\begin{array}{c} x∼\text{beta(}\alpha ,\beta \text{)}\text{with}\begin{array}{cc} \alpha & =1\\ \beta & =\frac{1-2C}{2C} \end{array} \end{array}$$

A less connected web will have more specialists, such that the distribution will skew more toward smaller range values. The range width for each species is then scaled to fall in (0, *n_i_*) so that it will never exceed the niche index, which is obtained by:

(2) $$r_{i}=x_{i}n_{i}$$

The predation range is then defined as $\left[c_{i}-\frac{r_{i}}{2},c_{i}+\frac{r_{i}}{2}\right]$ Thus, we can center their predation range using a uniform distribution, ($c_{i}\in U\left(\frac{r_{i}}{2},n_{i}\right)$), where *C_i_*is the center of the species dietary range. Species are considered nondiscriminatory beyond this, as in they consume all species within their dietary range. We discarded webs failing to satisfy certain requirements of biological realism, including the conditions that (a) all species are connected to the web either by predating or being predated on by other species; (b) every species has an autotroph in its food chain; (c) the web is connected, which ensures that our food web is not composed of several smaller, distinct food webs. We also confirm that (d) the generated web exhibits our desired level of connectance.

Once a food web has been created, the species are identified as autotrophs, invertebrates, or fishes (Yodzis & Innes, 1992). Autotrophs are identified by looking for the species that have no prey (i.e., basal species). Invertebrates and fishes are identified depending on the species trophic position under the assumption that herbivores are more likely to be invertebrates, and carnivores are more likely to be fishes (Romanuk, Hayward, & Hutchings, 2011). In particular, we assume that the three most apex predators are fish and that all the remaining species that are not autotrophs are invertebrates (following Tonin, 2011 and Martinez et al., 2012). Trophic position of each species is calculated using the short‐weighted trophic position (*T*; Williams & Martinez, 2000, 2004a), which is the average of two other trophic position metrics: the shortest trophic level to a basal species (*T1*) and the prey‐averaged trophic position (*T2*; see Supporting information Appendix for its calculation):

(3) $$T_{i}=\frac{T1_{i}+T2_{i}}{2},\forall \text{species}i.$$

The shortest trophic level (T1) is defined as the shortest path to a basal species plus 1:

(4) $$\begin{array}{cc} T1_{i} & =1+\min_{j\in \{ja_{\mathrm{ij}}=1\}}T1_{j} \end{array}$$

where *a_ij_* is a binary element from the species connection matrix.

Prey‐averaged trophic position for species *i* is 1 plus the average trophic position of all its prey:

(5) $$\begin{array}{cc} T2_{i} & =1+\sum_{j\in S}a_{\mathrm{ij}}\frac{T2_{j}}{P_{i}}\\ & =1+\sum_{j\in S_{\text{prey},i}}\frac{T2_{j}}{P_{i}}. \end{array}$$

where *P_i_* is the number of prey that species *i* consumes. We describe a computational shortcut to calculate *T2_i_* for each species in the Supporting information Appendix. The short‐weighted trophic position has been shown to be a better estimator of trophic position than *T1* or *T2* individually (Carscallen, Vandenberg, Lawson, Martinez, & Romanuk, 2012; Williams & Martinez, 2004a). Note that autotrophs (basal species) are assigned a trophic position of 1 in every trophic position metric which, is reflected in Equations (3) and (4).

### 2.2 Coupling life‐history and population dynamics in food webs

The first step to define the population dynamics of each species within the generated food webs is to determine how efficient species are at processing their food. We expand the methods used by Brose, Williams et al. (2006) to calculate species consumption rates based on species metabolic rates that are approximated by relative body size. The body sizes (accounted as body masses) of all species within the food web are related to the basal species. Therefore, the relative body masses of all the basal species are assigned a value of 1. Then, the relative body masses of the invertebrates and fishes are calculated assuming a constant body mass ratio between consumers and resources (the so‐called allometric ratio, *Z*), set to *Z* = 100 (Brose, Williams et al., 2006). Thus, the body mass is a simple function of trophic level Mass = *Z^T^* ^−1^, where 1 is subtracted from the trophic level to exclude basal species from the calculation (Brose, Williams et al., 2006).

Fish body mass is of importance not only because of dietary shifts but because metabolic rate per unit mass decreases with size. A school of large fish is more efficient at processing food than a school of small fish with the same biomass. In theory, this means that an ecosystem would be able to support a larger biomass of fish if the fish were larger. Kleiber's Law states that metabolic rates increase at a slower rate than body mass (Kleiber, 1975). While this law has been revised and modified many times, the underlying principle has held true (Ballesteros, Martínez, Luque, Lacasa, & Moya, 2014; Smil, 2000). For instance, a predator may be 100 times larger than its prey, but its metabolic rate is only 75 times that of its prey. Yodzis and Innes (1992) took advantage of this relationship to approximate how efficient the hypothetical organisms of this model convert energy from their food sources (Brose, 2008; Williams et al., 2007). Their calculations resulted in metabolic rate (*x_i_*) per unit of body weight (*M*) as:

(6) $$x_{i}=\left\{\begin{array}{cc} 0, & \text{forautotrophs}\\ 0.314M^{-0.15}, & \text{for invertebrates}\\ 0.88M^{-0.11}, & \text{forfish} \end{array}\right.$$

We use a deterministic algorithm to find the weight for new life‐history stages. From their weight, we can approximate their niche index so that we can fit them into the food web and their metabolic rates. We assign weights to three new, younger life‐history stages (*t* = 0,1,2) with a von Bertalanffy isometric growth curve (Pauly, 1980). Adults retain the original weight (*W*_max_) we assigned to each species, and we assume that is the life‐history stage (*t*_max_ = 3) and weight of maximum yield per recruit. The curvature of the von Bertalanffy curve is set as $K=\frac{3}{t_{\max}}$ (Froese & Binohlan, 2000), and we assume the adults reach $\frac{W_{\max}}{W_{\inf}}=0.9$ of their asymptotic weight.

(7) $$W_{t}=W_{\infty }{\left(1-e^{-K\left(t-t_{0}\right)}\right)}^{3}$$

**Figure 1:**
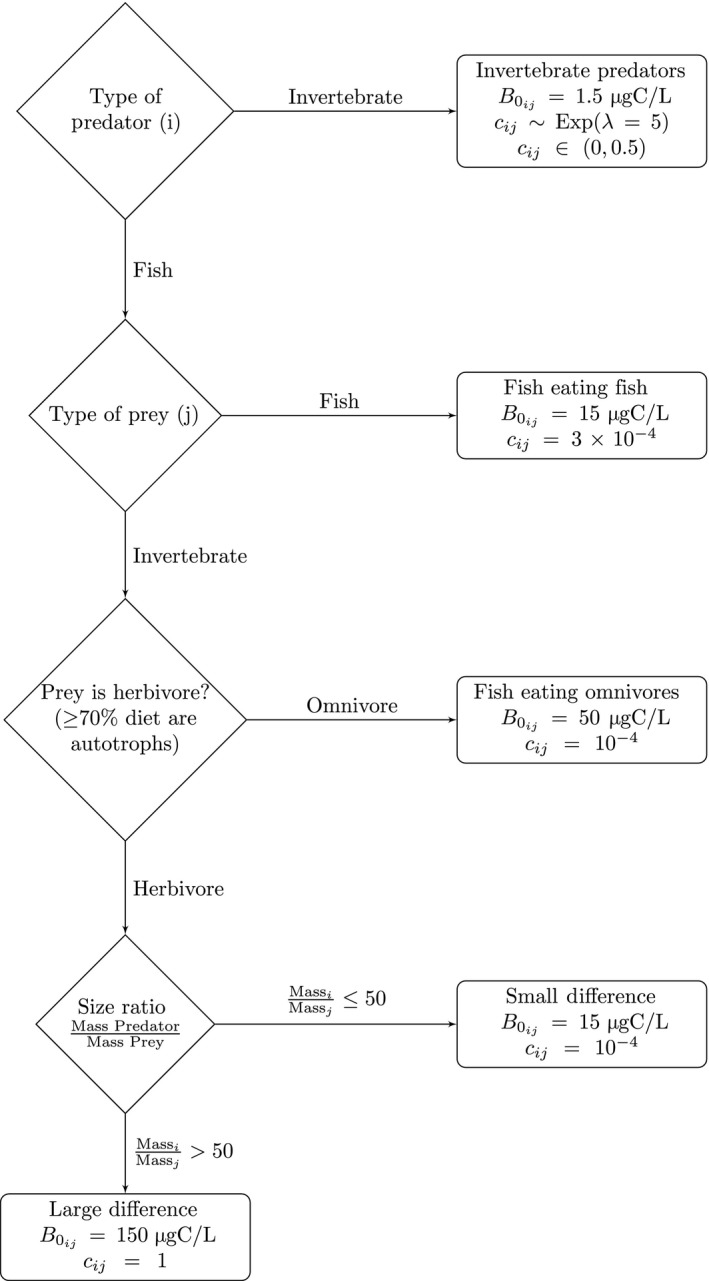
The half saturation constants ($B_{0_{\mathrm{ij}}}$) and competition coefficients (*c_ij_*) for predator *i* eating prey *j*. Figure and constants are reproduced from Tonin (2011) and Martinez et al. (2012)

The population dynamics of each species and life‐history stages within the food web can be described with ordinary differential equations (ODEs), which we use to simulate the biomass of each species. We modified the ATN model (Williams et al., 2007; Williams & Martinez, 2004b) to accommodate life‐history structure. The following equations from the ATN model show the growth for autotrophs (Equation (8) and consumers (Equation (9) during the growing season:

(8) $$\begin{array}{c} {\dot{B}}_{i}=\overset{\text{Intrinsic Growth}}{\overbrace{r_{i}\left(1-\sum_{j\in \text{Autotrophs}}\frac{B_{j}}{K}\right)B_{i}-}}\overset{\text{Loss to Grazing}}{\overbrace{\sum_{j\in \text{Consumers}}x_{j}y_{\mathrm{ji}}B_{j}\frac{F_{\mathrm{ji}}}{e_{\mathrm{ji}}}}} \end{array}$$

(9) $${\dot{B}}_{i}=\underset{\text{Metabolic Loss}}{\underbrace{-f_{m}x_{i}B_{i}}}+\underset{\text{Dietary Intake}}{\underbrace{\sum_{j\in \text{Resources}}f_{a}x_{i}y_{\mathrm{ij}}B_{i}F_{\mathrm{ij}}}}-\underset{\text{Loss to Predation}}{\underbrace{\sum_{j\in \text{Consumers}}x_{j}y_{\mathrm{ji}}B_{j}\frac{F_{\mathrm{ji}}}{e_{\mathrm{ji}}}}}$$

where $r_{i}$ is the intrinsic growth rate for autotroph *i*, *K* is the carrying capacity, *x_i_* is the metabolic rate (Equation (6)), *y_ij_* is predator *i*'s maximum consumption rate for prey *j*, *e_ij_* is the assimilation efficiency for *i* eating *j*, *f*_m_ is the fraction of assimilated carbon lost for maintenance, and *f_a_* is the fraction of assimilated carbon that contributes to growth. *F_ij_* is the normalized functional response:

(10) $$F_{\mathrm{ij}}=\frac{\omega_{\mathrm{ij}}B_{j}^{h}}{B_{0_{\mathrm{ij}}}^{h}+\sum_{k\in \text{consumer}}\left(c_{\mathrm{kj}}p_{\mathrm{ik}}B_{k}B_{0_{\mathrm{kj}}}^{h}\right)+\sum_{l\in \text{resources}}\left(\omega_{\mathrm{il}}B_{l}^{h}\right)}$$

where $\omega_{\mathrm{ij}}=1/P_{i}$ is the relative preference of species *i* on its prey *j*, *P_i_* is the total number of species *i*'s prey, *h* is the Hill exponent, $B_{0_{\mathrm{kj}}}$ is the half saturation density for *k* eating *j*, *c_kj_* is the predator interference of species *k* eating *j*, and *p_ik_* is the fraction of *i*'s resources that it shares in common with *k*. The values for these parameters are described in Table 1 and Figure 1.

**Table 1:** Model parameters

Variable	Description	Value	Unit	References
*S*	Number of species in original niche web	30	‐	Martinez et al. (2012)
*C*	Connectance	0.15	‐	Martinez et al. (2012)
*K*	Autotroph carrying capacity	540	µgC/L	Boit et al. (2012); Martinez et al. (2012)
*r*	Autotroph intrinsic growth rate	*r* ~ *N* (09,0.2) $r\in \left(0.6,1.2\right)$	*d* ^−1^	
*y_ij_*	Maximum consumption rate of predator *i* for prey *j*	10	*d* ^−1^	Boit et al. (2012)
*e_ij_*	Assimilation efficiency for *i* eating *j*	$\left\{\begin{array}{c} 0.45,\;j\;\text{is an autotroph}\\ 0.85,\;\text{otherwise} \end{array}\right.$	‐	Brose, Williams et al. (2006)
*h*	Hill Exponent	1.2	‐	
*f_a_*	Fraction of assimilated carbon that contributes to growth	0.4		Boit et al. (2012)
*f_m_*	Fraction of assimilated carbon lost for maintenance	0.1		Boit et al. (2012)

At the end of each growth season, the ODEs (Equations (8) and (9)) are paused so that fish may grow and reproduce. The biomass (*B_i_*) shifts between life‐history stages according to the following Leslie matrix:

(11) $$\left(\begin{array}{c} {\dot{B}}_{i}\\ {\dot{B}}_{i+1}\\ {\dot{B}}_{i+2}\\ {\dot{B}}_{i+3} \end{array}\right)=\left(\begin{array}{cccc} 0.1 & 0 & 0 & 0.9\\ 0.9 & 0.1 & 0 & 0\\ 0 & 0.9 & 0.1 & 0\\ 0 & 0 & 0.9 & 0.1 \end{array}\right)\left(\begin{array}{c} B_{i}\\ B_{i+1}\\ B_{i+2}\\ B_{i+3} \end{array}\right)$$

Essentially, this means that 90% of biomass grows to the next life‐history stage, while 10% remains in the previous stage. This choice was made to allow realistic phenotypic variability within the species, that is, most individuals grow from one age‐specific average size to the next age‐specific average size but a few individuals remain at the lower developmental stage (size) than expected based on their age. The highest (4th) life‐history stage reproduces and 90% of its biomass is transferred to the first life‐history stage as newborns. Notably, our formulation of the Leslie matrix allows the model to be applied to a broad range of ontogenetic developments, not only the most obvious application, which is aging from one age‐class to another (100% biomass transfer from one stage to another).

### 2.3 Simulation design and analyses

We investigated the model through systematic simulations to determine how inclusion of fish life‐history stages affects the food web, its structure, dynamics, and stability. The addition of life‐history structure for fishes changes multiple features of the food web. Introduction of life‐history stages involves the addition of new nodes and feeding links to the web; life‐history dynamics (growth from one life‐history stage to the next) alters the ways in which biomass is transferred within the food web.

To tease apart the relative roles of these components involved with the life‐history structures, we run 3 sets of simulations (hereafter denoted as “model types”). The first model type comprises an “original” or baseline web that does not include life‐history stages within species. That is, each species, including fish, is described through one single node in the food web. Model type 2 incorporates unlinked life‐history stages within each fish species. That is, each fish species is partitioned into life‐history stages, but these stages are not linked with one another through Leslie matrices. The new fish life stages are independent of each other, and biomass does not transfer through aging from one life‐history stage to another. In the ATN modeling sense, they can be considered as new species. While this model type is not biologically realistic, it is crucial for disentangling the effects of adding new nodes to the food web from the effect of life‐history dynamics. Model type 3 is an ATN model that incorporates life‐history stages that are linked to one another within each species using Equation (11).

To compare the three model types, we begin the simulations (500 for each model type) with the same initial conditions. In each simulation, the food web is allowed to stabilize for 200 years, after which the food web is either accepted or rejected, based on the rules detailed below. The dynamics of the food webs are then investigated across another 100‐year period. The burn‐in time and the investigated simulation period were chosen such that the node biomasses reached dynamic equilibriums and to allow sufficient temporal replication of the food web dynamics to capture short‐ and long‐term oscillations. Each year consists of 100 simulation time steps, representing a 100‐day growing season. Because our objective is to study the impact of fish life‐history stages, we choose among the stabilized food webs only those that contain at least one fish species or at least one fish life‐history stage (in model type 2). Life stages become extinct if their biomass is lower than 10^−^^6^ μg C/L, although fish species can be revitalized through aging, as biomass shifts from younger to older age classes. Thus, the final analyzed food webs contained from one to three fish species or, in the case of model 2, at least one fish life‐history stage.

We initially conducted a preliminary analysis on the probability of fish extinctions for each model type. For this preliminary analysis, we discarded only those food webs for which all fish became extinct. The remaining analyses were subjected to a more stringent constraint; at least one fish species must have persisted in every simulation run for a given model type for the web to be included. The robustness of the results to the choice of *Z* = 100 was explored by replicating the analyses with the values of *Z* generated randomly from lognormal distributions. The main difference was seen in the increased frequency of stable food webs when *Z* = 100, as compared to the scenario, where *Z* was randomly drawn from the lognormal distribution (results not shown). We used R version 3.3.2 (R Core Team, 2016) for all analyses, and the R library tidyverse (Wickham, 2017). We run the dynamic model with MATLAB version 2016 (The MathWorks).

## 3 RESULTS

**Figure 2:**
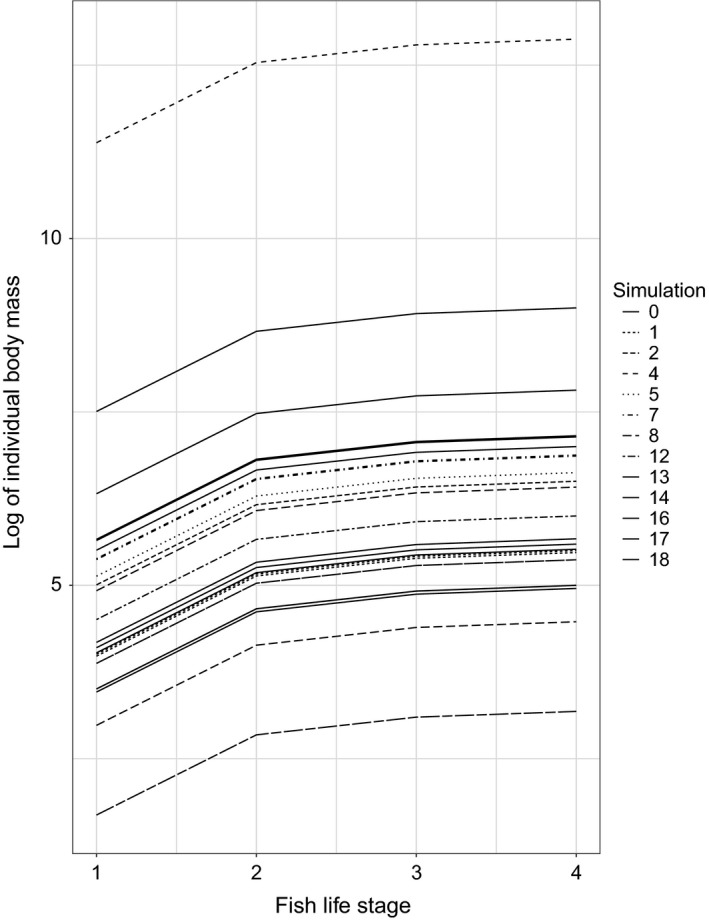
Von Bertalanffy growth curves for surviving fish in several simulated food webs. Each line type represents a different food web simulation. Each species has four life stages

One means of assessing the biological realism of the model was to examine the degree to which the model produced biologically realistic results. In this regard, our model produced realistic von Bertalanffy growth curves: mass is incomparable across simulations, but fish species within a single simulation tended to be in the same size range, as the weight ranges for fish species often overlap (Figure 2). The youngest life stage of the largest fish species was smaller than the oldest life stage of the smallest fish in 75.8% percent of the simulations.

A key criterion for the initial part of the analysis was to have the generic model achieve stability in overall fish biomass. Most (81.0%) of the simulations met this criterion, insofar as fish biomass stabilized in at least one of the experiments. A secondary criterion was that at least one fish species must achieve stability in each of the specific models; 24.4% of the simulations met this second criterion. Given that most simulations stabilized within 200 years, the initial 200 years were discarded and the remaining 100 years used for analysis.

**Figure 3:**
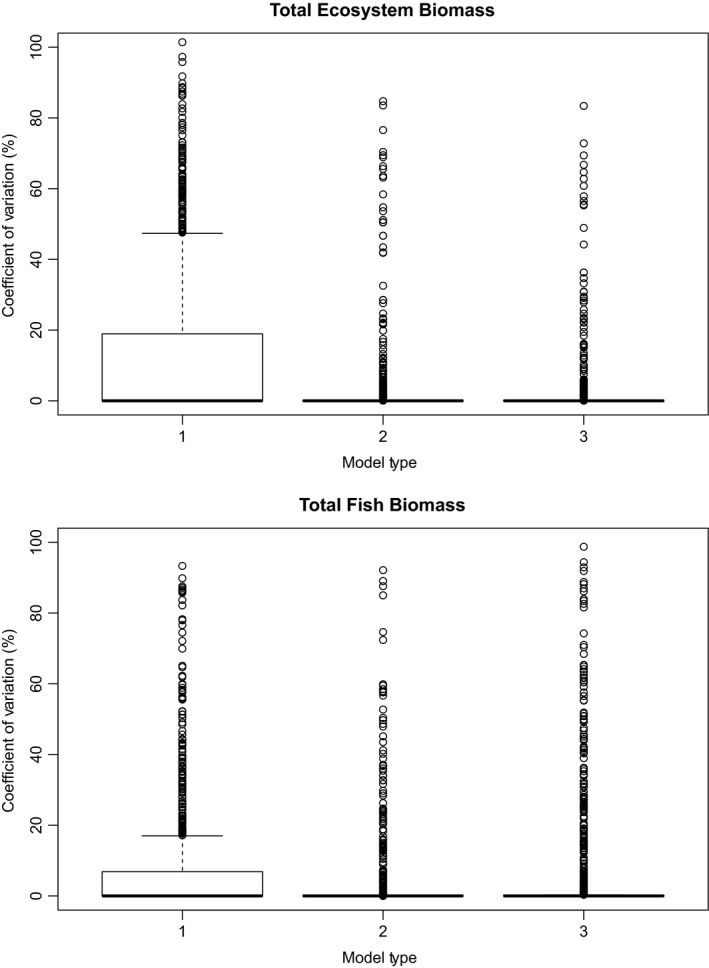
Boxplots of the coefficient of variation (CV) of the (a) total ecosystem biomass and (b) total fish biomass for each model type (CV's greater than 100 are not shown for clarity)

**Figure 4:**
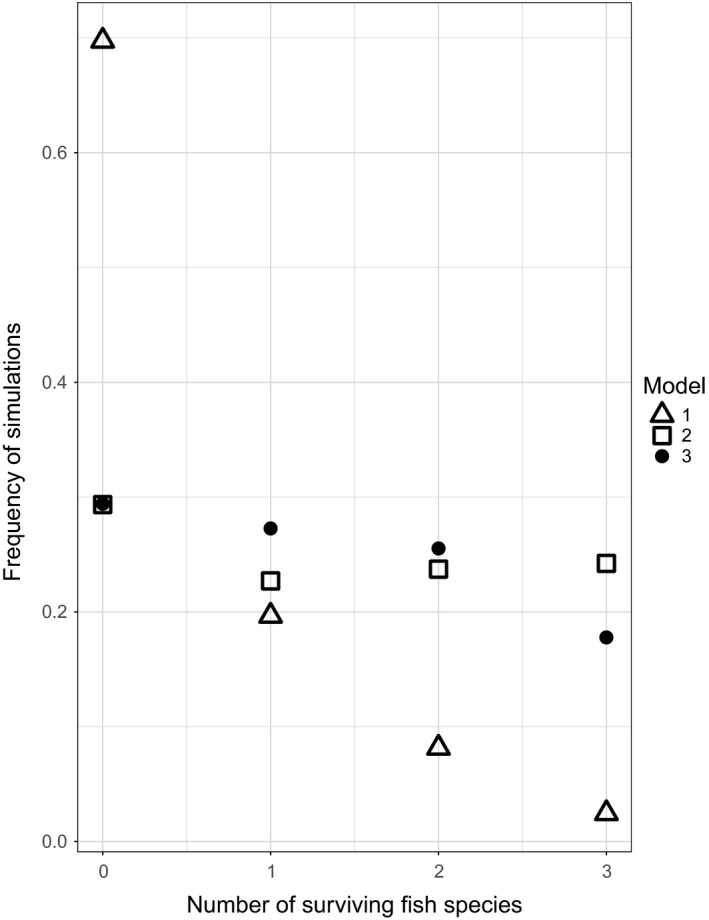
The frequency of simulations with 0, 1, 2, or 3 surviving fish species in each model. The different shapes indicate each model type: (1) the original ATN model (triangle), (2) extended unlinked model (square), and (3) the linked model (circle)

Neither the CV for total ecosystem biomass or total fish biomass (Figure 3) differed between the three model types. This result is supported by the frequency of the consecutive number of surviving fish species in each model (Figure 4). The model types that included new life stages were more likely to have at least one fish species survive, as well as having every fish species survive. There does not appear to be a difference between the linked model (model type 3) and unlinked model (model type 2). The unlinked model seems to have a more intermediate outcome, while linking the life histories seems to steepen the probability of consecutive extinctions.

**Figure 5:**
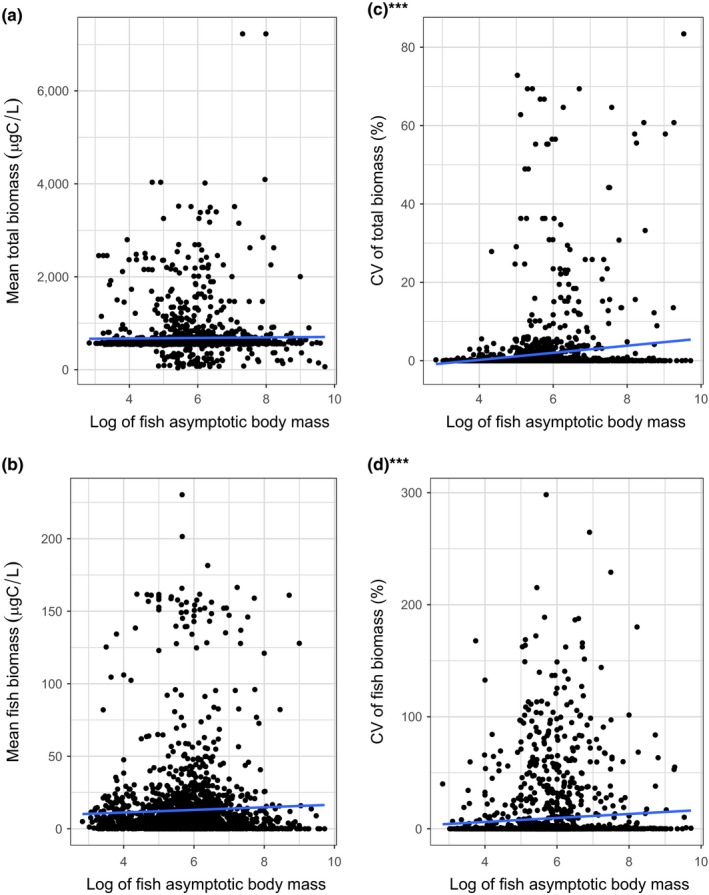
Mean and CV of biomass as a function of the asymptotic individual body mass for each surviving fish species. Panels (a) and (b) show the mean ecosystem biomass and mean biomass of the 1982 fish species, respectively. Panels (c) and (d) show their respective CV's. The blue lines represent linear regressions. These are significant for the CV of the total ecosystem biomass (panel c; *t* = 5.67, *df *= 1980, *p* < 0.001) and the CV of the fish biomass (panel d; *t* = 3.13, *df *= 1980, *p* = 0.002). Outliers with a mass larger than 10^10^ or CV greater than 800 were removed from the analysis

Simulation outputs are illustrated for the fully linked model (model type 3) (Figure 5; but see Supporting information figures in the electronic supporting materials for the analogous figures for model 1 and model 2). There is no correlation between fish size and mean total ecosystem biomass (*t* = 0.61, *df *= 1980, *p* = 0.544; Figure 5a) or mean fish biomass (*t* = 1.64, *df *= 1980, *p* = 0.102; Figure 5b). However, larger fish species are correlated with a higher CV for both the total ecosystem biomass (*t* = 5.67, *df *= 1980, *p* < 0.001; Figure 5c), and the CV of fish biomass (*t* = 3.13, *df *= 1980, *p* = 0.002; Figure 5d). Normality for each variable was confirmed using qqplots.

## 4 DISCUSSION

The present study opens new avenues of research in food web ecology by proposing a general framework to integrate life histories into the analysis of complex food webs. This framework extends the existing allometric trophic network (ATN) theory by incorporating life‐history structure. Using Williams and Martinez's (2000) niche model and the bioenergetics model (Yodzis & Innes, 1992) as starting points, we created life‐history structured ATN models. Firstly, we added additional life‐history stages, that is nodes, to each species. Secondly, we linked these stages together, such that juveniles grow into adults and then produce offspring. Through these additional biological mechanisms, we are able to evaluate the effect of life‐history dynamics on the function and stability of food webs. While we chose aquatic ecosystems, where fish species exhibit the life‐history dynamics, our theory can easily accommodate other types of systems and species exhibiting the life‐history dynamics through the broadly applicable Leslie matrix. Furthermore, our framework offers a systematic approach for disentangling the effects of increasing life‐history complexity in food‐wed models.

Here, we find that the addition of life‐history structure complexity significantly influences model outcomes, but that the linking of the stages within each fish species through a Leslie matrix alters the output of the unlinked model only marginally. For example, the addition of life‐history stages reduces variability in total ecosystem biomass, which we interpret as reflecting increased stability. Given that new, unlinked life‐history stages can be treated as new individual species, this finding is essentially equivalent to the conclusion that ecosystems which support greater numbers of fish species are more stable than ecosystems that support fewer fish species.

One potential pathway leading to the increased stability is via linking multiple size‐varying life‐history stages, which makes each species more dependent on a broader range of prey. In a sense, we are creating a scenario for increased species generalism by linking all the life stages and by making them less dependent on any one particular prey. On the other hand, we might also increase the extinction probability of a predator species if any one of its life‐history stages goes extinct. These nonviable life‐history stages may be partly responsible for why we failed to find a strong effect of linking the life stages together. Perhaps, if we ensured life‐history stage viability by assigning broader diets to each stage, we might have observed a larger effect of stage linkage. The linking of life‐history stages might also alleviate the predator‐induced mortality of certain prey species. If a fish predator is comprised of a wide variation of cohort sizes in its life‐history stages, the prey of any given stage may go through phases of intense predation when it is targeted by the largest cohort followed by a recovery period when the largest cohort is no longer preying on it.

The effects of increasing life‐history complexity on ecosystems were recently explored by Mougi (2017), who evaluated the effect of two life‐history stages on food webs that were randomly generated and whose dynamics were described by Lotka–Volterra population dynamics with linear functional responses. The author found that inclusion of two stages (rather than only one stage per species) increased the probability of persistence of complex food webs, while it decreased persistence for simpler food webs (Mougi, 2017). Based on the findings of the present study, we hypothesize that most of the effects that Mougi (2017) documented when adding life‐history structure might be attributable to an increase in food web size resulting from the addition of nonrandom nodes, rather than any intrinsic effect of life‐history structure. That said, our methods were quite different. The structure of our food webs was randomly generated by the niche model which has been demonstrated to generate realistic structures when compared with empirical food webs (Williams & Martinez, 2000). Moreover, the parameters used in our population dynamics come from allometric relations well supported by empirical studies (Brown, Gillooly, Allen, Savage, & West, 2004; Enquist, West, Charnov, & Brown, 1999). Additionally, the functional responses used in our model incorporate consumption saturation that has been demonstrated to be much more biologically meaningful than linear functional responses (Holling, 1959). Therefore, we think our theory is a substantial advance after the contribution of Mougi's (2017) work given that our theory is better supported empirically. Finally, we applied an annual Leslie matrix to model growth from one life stage to the next, while Mougi (2017) incorporated a continuous growth model directly into the differential equations. We used four life stages for three species, while he used two life stages for various proportions of the community.

Future research should deal with some of the limitations of the theory we present here. Our application of the von Bertalanffy growth model lends increased biological realism in terms of body mass and consequently metabolic rate. However, the species all have identical life histories (exactly four life stages, identical age‐specific probabilities of maturity, and the same age‐specific fecundity). It might be worth exploring alternative life spans and life‐history strategies in future model formulations. Moreover, our results suggest that it would be instructive to increase life‐history complexity in the models that explore the impacts of fishing on the target ecosystems (e.g., Kuparinen, Boit, Valdovinos, Lassaux, & Martinez, 2016). From an ecosystem‐based management perspective, it would be important to examine how size‐selective fishing mortality, which would differentially affect some species and life‐history stages more than others, influences species persistence and ecosystem functionality.

While the focus of our study was on aquatic food webs, several other applied questions leveraging the relevance of life‐history dynamics in food webs and ecological networks in general can benefit from the theory developed here. Such applications of the theory might include biological control, ecosystems services such as pollination, and responses of ecosystems to various types of anthropogenic perturbations.

## DATA ARCHIVING

Codes and simulation outputs can be found in Dryad (http://dx.doi.org/10.5061/dryad.1hd6dg7).

## AUTHOR CONTRIBUTIONS

SB, JAH, and AK designed the study. SB, FSV, and AK developed the model. SB conducted the simulations with assistance from FSV. SB analyzed the results with assistance from AK and JAH. SB, FSV, JAH, and AK wrote the article.
