# CORRIGENDUM

**DOI:** 10.1002/ece3.7270

**Published:** 2021-03-20

In “The role of fish life histories in allometrically scaled food‐web dynamics,” which was published in volume 9 issue 6, March 2019, in the first sentence of the acknowledgments section, the stated project number for COMPLEX‐FISH was wrong:

(COMPLEX‐FISH 400820 to A.K.).

The correct project number should be:

(COMPLEX‐FISH 770884 to A.K.).
