# Supplementary material for: The role of fish life histories in allometrically scaled food‐web dynamics
Source: Ecol Evol. 2019 Feb 21;9(6):3651–60. doi: 10.1002/ece3.4996 (PMC6434563; doi:10.1002/ece3.4996)

ELECTRONIC APPENDICES

Appendix A. Supporting mathematics

- 1. Derivation Of *β* For The Beta Function

In order to get a beta distribution of the prey range values (*r­_i_*), we used a beta distribution with the parameters specified in equation 1 in the main text. The value for *β* is set so that the expected value for a species range is the connectance (*C*):

$$\begin{matrix} C=E(r_{i}) & =E(n_{i}x_{i}) \\ & =E(n_{i})E(x_{i})\text{, since }x_{i}\text{ and }n_{i}\text{ are independent} \\ & =\frac{1}{2}E(x_{i})\text{, since }n\sim\text{U}(0,1) \\ & =\frac{1}{2}\frac{\alpha}{\alpha+\beta}\text{, since }x\sim\text{beta}(\alpha,\beta) \\ 2C(\alpha+\beta) & =\alpha\\ 2\beta C & =\alpha-2\alpha C \\ \beta& =\frac{\alpha(1-2C)}{2C}\text{, and since }\alpha=1 \\ \beta& =\frac{1-2C}{2C} \end{matrix}$$

- 1. Prey-Averaged Trophic Position

Prey averaged trophic position for a single species can easily be calculated using equation 3 in the main text if you have already established the trophic positions of all of its prey, but this is more complicated for webs with loops. The earlier equation (eqn 3 in the main text) is certainly more intuitive, but for practical purposes we will present an equivalent way of solving for prey averaged trophic position using matrix algebra (Levine, 1980).

The species connection matrix is the primary way of conveying food web information, but here we will use a transition matrix instead. As you may recall, the connection matrix has simple binary links between species, indicating the presence or absence of predation. Transition matrices, on the other hand, also convey how much a consumer relies on each species in its diet. Each element *q_ij_* of the transition matrix, *Q*, is defined as the fraction that species *j* consists of in consumer *i*'s diet:

$$\begin{matrix} q_{ij} & =\left\{ \begin{matrix} \text{Fraction of consumer i's prey that is species j,} & \text{ if species i has prey;} \\ 0, & \text{ if species i is an autotroph.} \end{matrix} \right. \\ & =\left\{ \begin{matrix} \frac{a_{ij}}{\sum_{j\in S} a_{ij}}, & \text{ if }\sum_{j\in S} a_{ij}\neq0; \\ 0, & \text{ if }\sum_{j\in S} a_{ij}=0. \end{matrix} \right. \end{matrix}$$

Transition matrices (*Q*) are powerful because they describe how energy flows through a food web. By taking the *k*th power of the matrix (*Q^k^*), you can determine what proportion of a consumer's diet came through exactly k trophic levels from each resource. In other words, each element *b_ij_* of *B=Q^k^* describes how much energy came through a food chain of length k from resource *j* for consumer *i*. So element *c_ij_* in the geometric series

$$C=\sum_{k=0}^{\infty} Q^{k}=I+Q+Q^{2}+Q^{3}+\ldots$$

describes the average food chain length from resource *j* to consumer *i*, weighted by its importance to the consumer's diet (Orponen, 2005). So the sum of each row is equivalent to our desired prey-averaged trophic position (equation 3 in the main text).

Conveniently, we can easily rearrange matrix geometric series:

$(I-Q)^{-1}=I+Q+Q^{2}+Q^{3}+\text{…}$

Which holds provided that $\lim_{k\to\infty} Q^{k}=0$ (Watson, 2015). This is the case if all species have an autotroph in their food chain, so this calculation works for all biologically realistic food webs.

The end result is that we can use the following, computationally efficient, equation to solve for the prey averaged trophic position (*T2_i_*) for species *i*:

$$\begin{aligned} T2=\left[ \begin{aligned} T2_{1} \\ T2_{2} \\ \vdots\\ T2_{S} \end{aligned} \right]=\left( I-Q \right)^{-1}\left[ \begin{aligned} 1 \\ 1 \\ \vdots\\ 1 \end{aligned} \right] \end{aligned}$$

- 1. Formal Proof

We will present a proof of the equivalence. See Levine (1980) for an alternative proof.

This part of the proof is analogous to the convergence of the geometric series $\frac{1}{1-z}=\sum_{i=0}^{\infty} z^{i}$, for complex number *z* with $\left| z \right|<1$ (Watson, 2015). Let:

$$C_{k}=\sum_{i=0}^{k} Q^{i}=I+Q+Q^{2}+Q^{3}+\text{…}+Q^{k}$$

So when you left-multiply by Q:

$$QC_{k}=\sum_{i=1}^{k+1} Q^{i}=Q+Q^{2}+Q^{3}+\text{…}+Q^{k}+Q^{k+1}$$

So

$$\begin{matrix} C_{k}-QC_{k} & =\left( I+Q+Q^{2}+Q^{3}+\text{…}+Q^{k} \right)-\left( Q+Q^{2}+Q^{3}+\text{…}+Q^{k}+Q^{k+1} \right) \\ (I-Q)C_{k} & =I-Q^{k+1} \end{matrix}$$

And similarly, if you right-multiply by Q:

$$C_{k}(I-Q)=I-Q^{k+1}$$

So, since $\lim_{k\to\infty} Q^{k+1}=0$:

$$\begin{matrix} C(I-Q) & =(I-Q)C=\lim_{k\to\infty} \left( I-Q^{k+1} \right)=I \\ C & =(I-Q)^{-1} \end{matrix}$$

Appendix B: Codes and data deposited in Dryad

**B.1 Main files:**

START_RunCluster_2018May03_1.zip

We modified a the ATN model from Tonin (2011) and Martinez et al. (2012) by extending it to include life history structure. We use MATLAB version 2016b (The MathWorks). The main file within is called START_RunCluster.m.

read_me.docx

A description of the files in the ATN model (START_RunCluster_2018May03_1.zip).

reformat_data.R

This R code inputs the simulation output and reformats it into tidy data (Wickham 2014), as contained in clean_2018May03_1.zip, clean_phase1_2018May03_1.zip, or clean_phase2_2018May03_1.zip. It also creates colnames_clean_2018May03_1.txt, which stores the column names for the data sets.

Appendix_Analysis.R

This R code does all the analyses described in the main text. It requires clean_phase2_2018May03_1.txt, which is contained in clean_phase2_2018May03_1.zip, and colnames_clean_2018May03_1.txt.

clean_phase1_2018May03_1.zip

This contains the compressed text file for the first phase (200 years) of the simulation during which food web dynamics reach the equilibrium.

clean_phase2_2018May03_1.zip

This contains the compressed text file for the second phase (100 years) of the simulation, after the food web dynamics have reached equilibrium. This is the data set we use in the analyses (Appendix_Analysis.R), as it is much smaller than the complete data set.

colnames_clean_2018May03_1.txt

This contains the column names for all the data sets. This file is called by Appendix_Analysis.R.

**Appendix C: Supporting figures**

**Fig. S1** Mean and CV of biomass as a function of the asymptotic individual body mass for each surviving fish species in model 1. Panels (a) and (b) show the mean ecosystem biomass and mean biomass of the 1405 fish species, respectively (N=1405 fish species across all simulations). Panels (c) and (d) show their respective CV’s. The blue lines represent linear regressions. These are significant for the mean of the fish biomass (panel b; t=4.04, df=1403, p<0.001) and the CV of the total ecosystem biomass (panel c; t=9.06, df=1403, p<0.001). Outliers with a mass larger than 1010 or CV greater than 800 were removed from the analysis.

**
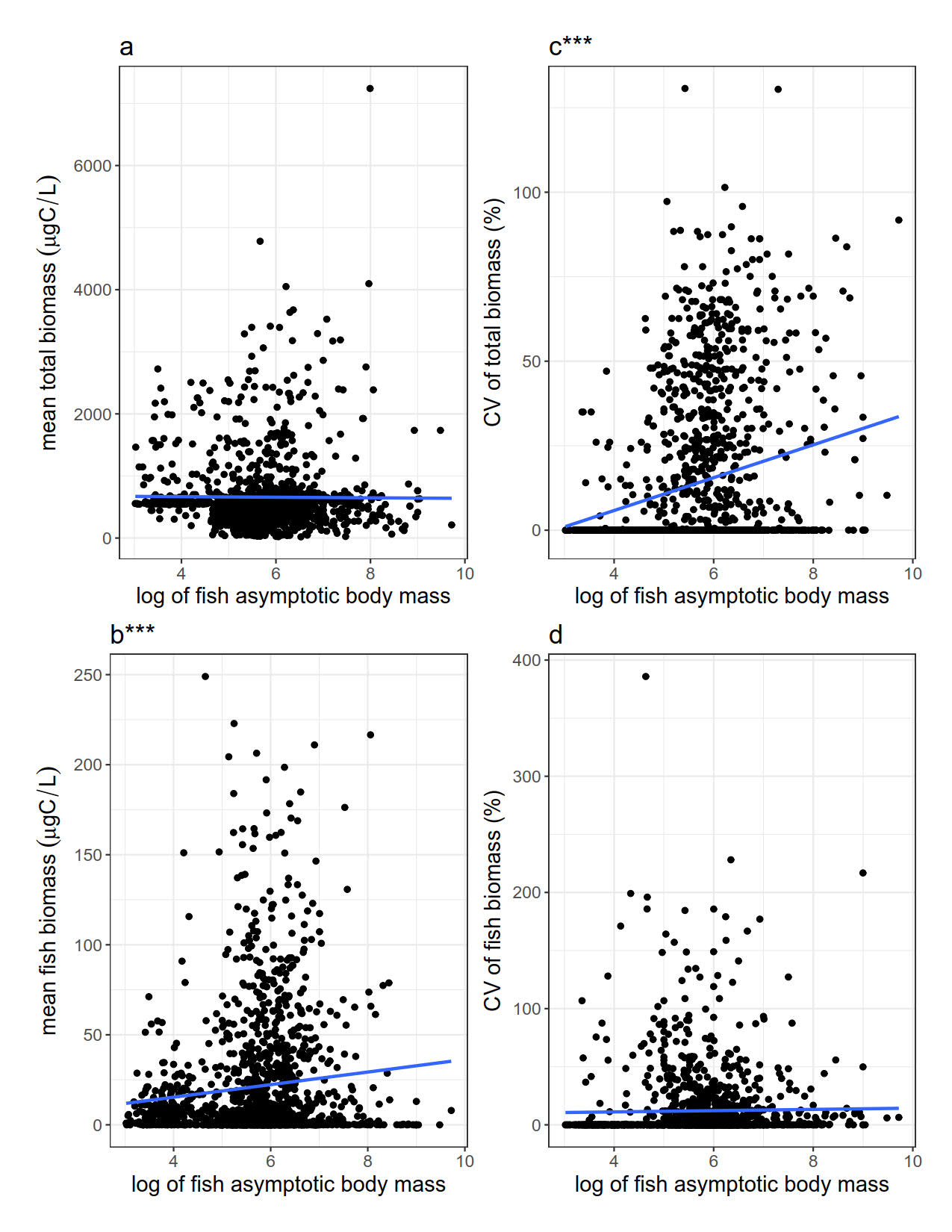
**

**Fig. S2** Mean and CV of biomass as a function of the asymptotic individual body mass for each surviving fish species in model 2. Panels (a) and (b) show the mean ecosystem biomass and mean biomass of the 2157 fish species, respectively (N=2157 fish species across all simulations). Panels (c) and (d) show their respective CV’s. The blue lines represent linear regressions. These are significant for the mean of the total ecosystem biomass (panel a; t=2.34, df=2155, p=0.019) and the CV of the total ecosystem biomass (panel c; t=6.45, df=2155, p<0.001). There are also nonsignificant trends for the mean of the fish ecosystem biomass (panel b; t=1.95, df=2155, p=0.051) and the CV of the fish ecosystem biomass (panel d; t=1.75, df=2155, p=0.08). Outliers with a mass larger than 1010 or CV greater than 800 were removed from the analysis.


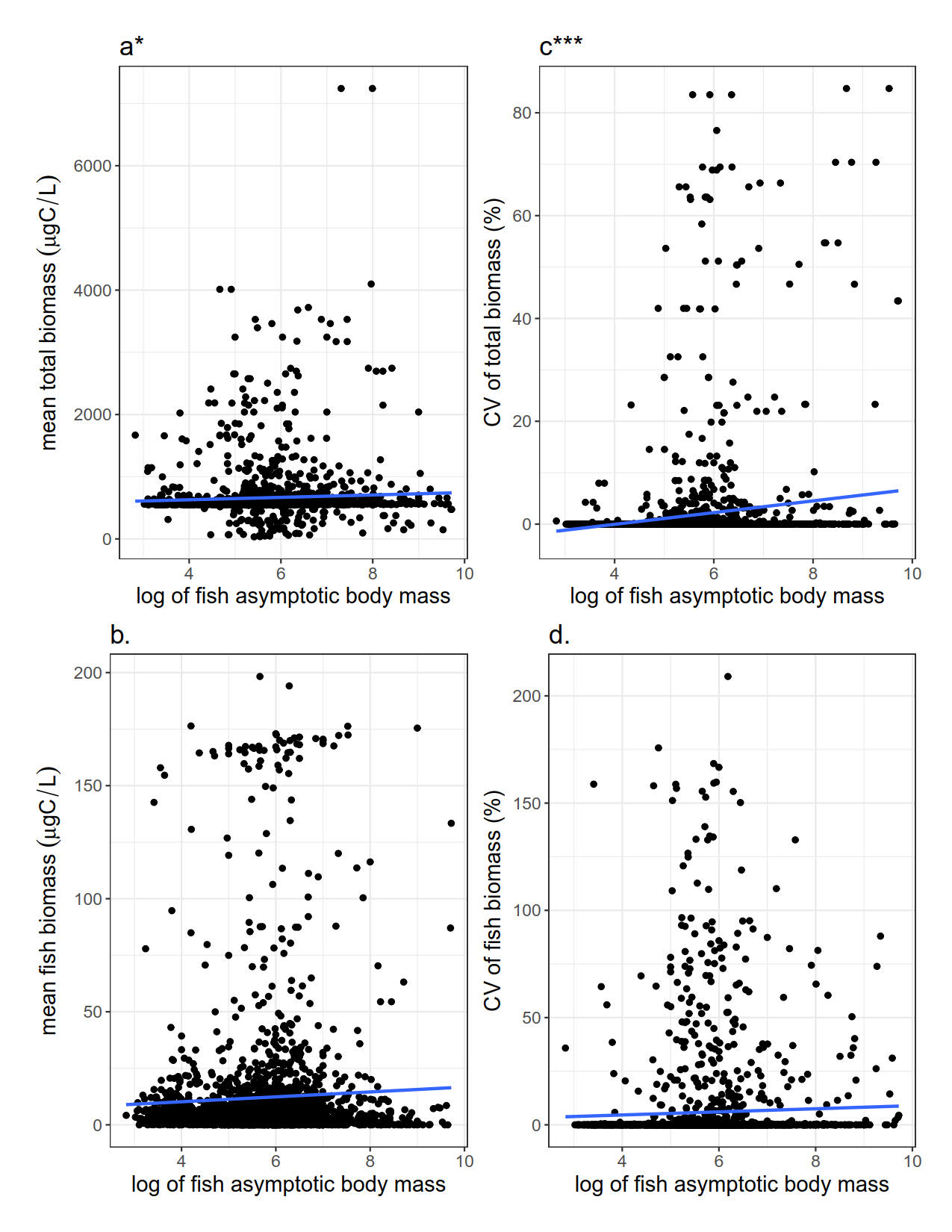

Supplement: Supplementary file 1 [file ECE3-9-3651-s001.docx]
